# Supplementary material for: Effect of Gestational Age at Fetoscopic Laser Photocoagulation on Perinatal Outcomes for Patients with Twin–Twin Transfusion Syndrome
Source: J Clin Med. 2023 Feb 28;12(5):1900. doi: 10.3390/jcm12051900 (PMC10003859; doi:10.3390/jcm12051900)
Supplement: Supplementary file 1 [file jcm-12-01900-s001.zip › jcm-2172884-supplementary.pdf]

Table S1: TTTS received FLP after 2015 March with and without Solomon technique

	With Solomon (N=12)	Only SLPCV (N=87)	p
At least fetal survival (N)	11	74	1.0
Two survival (N)	7	54	1.0
Maternal age at FLP (year)	31.4	32.4	0.51
PROMs after FLP (N))	1	9	1.0
GA at FLP (weeks)	19.5	20.1	0.062
GA at delivery (weeks)	31.4	32.4	0.15

TTTS: twin-twin transfusion syndrome

FLP: fetoscopic laser photocoagulation

Table S2: TTTS with and without cerclage after FLP

	Without cerclage (N=169)	With cerclage (N=28)	p
At least fetal survival (N)	147 (87.0%)	17 (60.7%)	0.002
Two survival (N)	104 (61.5%)	13 (46.5%)	0.149
PROMs after FLP (N))	18 (10.7%)	8 (28.6%)	0.016
GA at FLP (weeks)	20.5	21.4	0.077
GA at delivery (weeks)	32.0	28.4	0.007

TTTS: twin-twin transfusion syndrome

FLP: fetoscopic laser photocoagulation
